# Development and implementation of a clinical decision support system-based quality initiative to reduce central line-associated bloodstream infections

**DOI:** 10.1017/cts.2024.566

**Published:** 2024-09-23

**Authors:** Michelle C. Spiegel, Andrew J. Goodwin

**Affiliations:** Division of Pulmonary, Critical Care, Allergy, and Sleep Medicine, Department of Medicine, Medical University of South Carolina, Charleston, SC, USA

**Keywords:** Clinical decision support systems, quality improvement, implementation science, critical care, healthcare-acquired infections

## Abstract

**Background:**

Central venous lines (CVLs) are frequently utilized in critically ill patients and confer a risk of central line-associated bloodstream infections (CLABSIs). CLABSIs are associated with increased mortality, extended hospitalization, and increased costs. Unnecessary CVL utilization contributes to CLABSIs. This initiative sought to implement a clinical decision support system (CDSS) within an electronic health record (EHR) to quantify the prevalence of potentially unnecessary CVLs and improve their timely removal in six adult intensive care units (ICUs).

**Methods:**

Intervention components included: (1) evaluating existing CDSS’ effectiveness, (2) clinician education, (3) developing/implementing an EHR-based CDSS to identify potentially unnecessary CVLs, (4) audit/feedback, and (5) reviewing EHR/institutional data to compare rates of removal of potentially unnecessary CVLs, device utilization, and CLABSIs pre- and postimplementation. Data was evaluated with statistical process control charts, chi-square analyses, and incidence rate ratios.

**Results:**

Preimplementation, 25.2% of CVLs were potentially removable, and the mean weekly proportion of these CVLs that were removed within 24 hours was 20.0%. Postimplementation, a greater proportion of potentially unnecessary CVLs were removed (29%, *p* < 0.0001), CVL utilization decreased, and days between CLABSIs increased. The intervention was most effective in ICUs staffed by pulmonary/critical care physicians, who received monthly audit/feedback, where timely CVL removal increased from a mean of 18.0% to 30.5% (*p* < 0.0001) and days between CLABSIs increased from 17.3 to 25.7.

**Conclusions:**

A significant proportion of active CVLs were potentially unnecessary. CDSS implementation, in conjunction with audit and feedback, correlated with a sustained increase in timely CVL removal and an increase in days between CLABSIs.

## Introduction

Central venous lines (CVLs) are frequently utilized in the care of critically ill patients. However, despite their advantages, their use is associated with approximately 30,000 central line-associated bloodstream infections (CLABSIs) in the U.S. annually [1]. These healthcare-associated infections (HAIs) are associated with poorer patient outcomes, including increased mortality and hospital length of stay [2,3] and they increase hospital costs by more than $48,000 per episode [4]. Thus, reducing preventable CLABSIs [5] is critically important and has been a focal point of hospital quality initiatives.

While U.S. hospitals had significantly reduced the incidence of CLABSIs in recent years, this progress was rapidly reversed during the COVID-19 pandemic [1,5–7]. There were multiple potential contributors for this reversal, including patient-related challenges (e.g. maintaining CVL dressings in patients requiring prone positioning) and healthcare worker-related challenges (e.g. capacity strain, staffing shortages [3], and burnout [6]) that likely exacerbated underlying vulnerabilities in safety culture [6]. Unfortunately, burnout has persisted postpandemic [8], and attrition of veteran care team members has led to ongoing staffing shortages and higher proportions of inexperienced team members [9] who are less familiar with HAI prevention measures. Accordingly, there is a need to refocus on quality and safety efforts and to develop strategies for their successful implementation in the current healthcare environment.

Prolonged and unnecessary CVL utilization is an important, but underemphasized, contributor to CLABSIs [10–12]. Identified barriers to timely CVL removal include inconsistent communication between care team members, the expanding cognitive burden of reviewing large amounts of data for each patient, competing priorities regarding device de-escalation, lack of awareness of the CVL and the indications for its removal, and difficulties finding CVL-relevant data [10,13,14]. Additionally, though recent studies [15–18] have suggested the safety of peripheral vasopressor administration under certain conditions [17], clinician adoption of this practice has been variable [18]. Clinical decision support systems (CDSSs) have emerged as potential tools for improving the quality of care [19] and promoting infection prevention [20], and they may provide a mechanism for addressing several of these barriers. However, thoughtful consideration of CDSS design is critical, as perceived negative impacts on efficiency, inadequate patient specificity, overly simplistic logic, and inappropriate triggering have historically hindered CDSS adoption [21–23].

This initiative sought to develop, implement, and evaluate a novel CDSS within an electronic health record (EHR) to quantify the prevalence of potentially unnecessary CVLs and improve timely removal of these devices at our institution. We hypothesized that, on average, at least 20% of active CVLs across adult intensive care units (ICUs) were potentially unnecessary, and that implementation of a CDSS would (1) increase the average proportion of these potentially unnecessary devices removed each week and (2) decrease the incidence of CLABSIs.

## Methods

### Design and setting

We conducted an implementation science-informed quality improvement initiative at the Medical University of South Carolina (MUSC), an 820-bed, academic, and quaternary care hospital with six adult ICUs. Components of the initiative included: (1) evaluating the effectiveness of pertinent existing CDSSs, (2) targeted clinician education, (3) developing and implementing a novel CDSS within our EHR to identify potentially unnecessary CVLs, and (4) reviewing EHR and other institutional data to compare rates of removal of potentially unnecessary CVLs, device utilization rates, and CLABSI incidence pre- and postimplementation. Data from all adult ICU patients for whom a CVL was utilized between September 2022 and August 2023 were included in the analysis. The primary outcome measure was the average weekly proportion of potentially unnecessary CVLs removed within 24 hours of identification, while CLABSI incidence and days between CLABSIs were secondary outcomes. The Quality Implementation Framework [24] was utilized in conjunction with plan, do, study, act (PDSA) cycles to guide the development and implementation of our intervention (Supplemental Table 1). This initiative was designated as quality improvement by the MUSC institutional review board and considered exempt from review. The findings of this intervention were reported in accordance with the SQUIRE 2.0 guidelines.

### Initial contextual considerations

#### Institutional CLABSI trends

Between July 2016 and June 2021, Adult ICUs at MUSC averaged 12.6 CLABSIs/year (0.95 CLABSIs/1,000 CVL days). Between July 2021 and June 2022, the incidence increased to 35 CLABSIs (2.06 CLABSIs/1,000 CVL days). Dates and unit locations of CLABSIs are internally reported on MUSC’s Tableau infection prevention dashboard (Seattle, WA, USA).

#### Evaluating the existing CDSS and CDSS infrastructure

MUSC has utilized Epic (Epic Systems, Verona, WI, USA) EHR since 2014. Prior to the present initiative, the EHR deployed daily interruptive alerts upon opening the charts of those patients with documentation of CVLs in place for > 72 hours (Supplemental Figure 1) as a strategy to promote de-escalation. This hard-stop alert was deployed for all physicians and advanced practice providers (APPs) opening the chart, regardless of clinical role and the patient’s potential ongoing need for the device, and clinicians were required to make a removal decision prior to reviewing the chart to determine necessity. Over a 7-month period (2/2022 through 8/2022), the CVL alert displayed 14,294 times and generated only 401 orders for CVL removal (97.2% of alerts overridden). As CVL days were the only trigger for the alert, it was not possible to confirm the appropriateness of the overrides. All CDSS’ must be approved by the institution’s CDSS Steering Committee prior to implementation.

#### ICU team composition and rounding practices

ICU clinician teams at MUSC include one attending physician, one fellow physician, APPs, residents, and medical students. Nursing participation during patient rounds varies by ICU. All ICUs have multiple computer workstations on wheels available to review EHR data during rounds.

### Developing the novel clinical decision support system

After receiving CDSS Steering Committee approval to build the CDSS, a multidisciplinary group of two pulmonary and critical care medicine (PCCM) physician investigators, three ICU nurses, and one ICU pharmacist reviewed institutional policies and medication and infusion administration guidelines to compile a list of appropriate indications for CVLs by type: double/triple/quad lumen catheters, hemodialysis (HD) catheters, and peripherally inserted central catheters (PICCs). Each of these indications was then mapped, when possible, to a queryable location within a patient’s Epic chart. The final list of CVL indications and their Epic correlates are listed in Table 1.

**Table 1.** Appropriate indications for CVL by catheter type with epic correlate

Catheter Type	Indication	Lookback	Epic Correlate
Double/triple/quad lumen CVL, PICC	Vasopressor requirement exceeding institutional peripheral vasopressor dosing limits	24 hours	Medication administration record (MAR)
	Receipt of TPN	48 hours	Intake/output flowsheet
	Receipt of TTM via internal cooling	24 hours	Induced hypothermia assessment flowsheet
	Use of pulmonary artery catheter to guide mechanical circulatory support	24 hours	Adult PCS body system flowsheet
	Administration of vesicant infusion (>3% hypertonic saline, calcium chloride, calcium gluconate, dextrose 20%)	24 hours	MAR
	Actively receiving chemotherapy (PICC-only)	N/A	Active oncology treatment plan
Hemodialysis Catheter	Receipt of renal replacement therapy	72 hours	CRRT, dialysis treatment flowsheets
	Receipt of therapeutic plasma exchange or exchange transfusion	72 hours	Hemapheresis flowsheet

CVL = central venous line, PICC = peripherally-inserted central catheter, TPN = total parenteral nutrition, TTM = targeted temperature management, CRRT = continuous renal replacement therapy, MAR = medication administration record.

**Figure 1.**
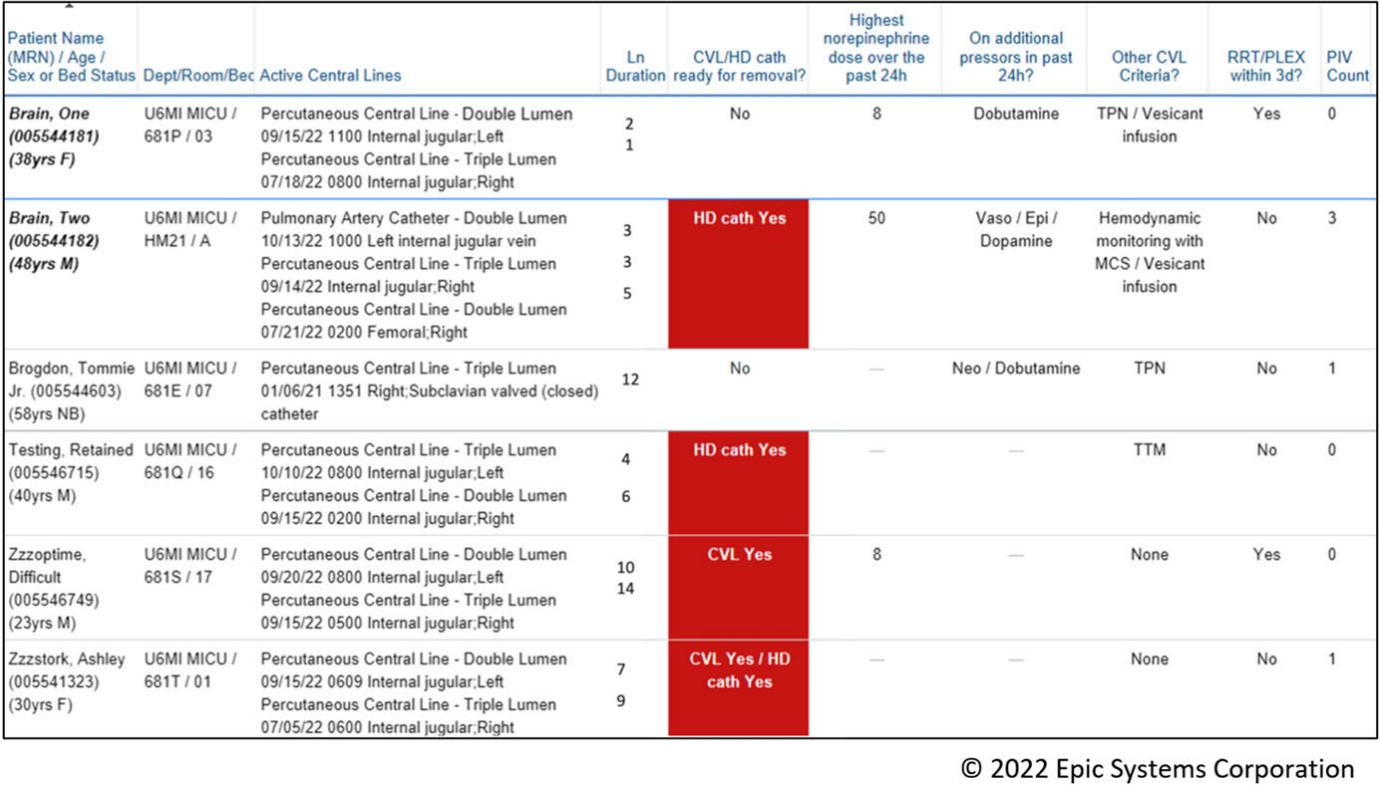
Prototype of ICU QI inpatient list. CVL = central venous line, HD cath = hemodialysis catheter, vaso = vasopressin, epi = epinephrine, neo = phenylephrine, TPN = total parenteral nutrition, MCS = mechanical circulatory support, TTM = targeted temperature management, PIV = peripheral intravenous access.

Once the criteria for CVL appropriateness were established, the physician investigators, with the assistance of an informaticist, developed the CDSS within Epic. Recognizing the limitations of interruptive pop-up alerts [25–27] and the importance of integrating CDSSs into existing workflows [28], the group built noninterruptive inpatient quality improvement (QI) lists to provide the CDS (Figure 1). These lists identified all active CVLs for a given team or unit on a single screen, and displayed, across multiple columns, the data that is typically needed for removal decisions (Table 1). For double/triple/quad lumen catheters and PICCs (identified in the CDSS as “CVL”), this included the highest dose of norepinephrine administered, whether the patient required additional vasopressors and whether additional CVL criteria were met (total parenteral nutrition, targeted temperature management using internal cooling catheter, use of a pulmonary artery catheter to guide mechanical circulatory support, vesicant infusions, active chemotherapy plan for PICCs). For HD catheters (identified in the CDSS as “HD cath”), the lists displayed whether the patient was undergoing renal replacement therapy, therapeutic plasma exchange, or exchange transfusion. Using human factors engineering principles [29–31], a device was highlighted in red if it was in place for > 24 hours and potentially eligible for removal based upon an absence of identified indications. Catheter duration, in days, was included on the lists but was not incorporated into the logic structure, as time-triggered replacement of CVLs does not reduce the risk of CLABSIs [32]. The number of currently placed peripheral IVs was also included to facilitate preparations for CVL removal but was not incorporated into the logic structure. Service team-based lists were created for each physician/APP ICU team, as patients on a given team may span multiple geographic ICUs, while unit-based lists were created for nurses.

### Preparing for implementation

To prepare for implementation of the inpatient lists, the physician investigators met with the physician medical directors and nurse managers for each of the six adult ICUs – two medical ICUs (MICU, specialty MICU (SMICU)); one combined medical/surgical ICU (MSICU); one combined medical cardiac/cardiothoracic surgical ICU (CVICU); one surgical/trauma/burn ICU; and one neurosciences ICU (NSICU) – to describe the intervention and its rationale, demonstrate the lists, and solicit feedback. The institution’s peripheral vasopressor policy (Supplemental Appendix) was also reviewed during these meetings. These physician and nurse leaders then served as champions to further disseminate the presented information to the relevant care team members (physicians, APPs, nurses) for their respective ICUs. The physician investigators prepared the three ICUs for which their division provides care (MICU, SMICU, MSICU) by providing education to all PCCM faculty and fellows, and by aligning the required annual fellowship and APP quality improvement projects with the goals of this intervention. They also met with additional nursing groups across the ICUs when requested by unit leadership. The day prior to implementation, one of the physician investigators distributed, via email, a “tip sheet” explaining the intervention and providing instructions for saving the appropriate inpatient lists as quick-access “favorites” to the physician medical directors, nurse managers, all ICU APPs, and all PCCM physicians with a request for further distribution to additional care team members. The preexisting CVL CDSS was de-implemented prior to implementation of the inpatient lists.

### Data collection and statistical analysis

Data for overall CVL utilization (CVL days per patient days) over time were collected through MUSC’s Tableau infection prevention dashboard. To facilitate further evaluation of all adult ICU patients with CVLs, an Epic Reporting Workbench report, which included the same columns as the inpatient lists, was built and queried daily at 0800. One of the PCCM physician investigators reviewed the report each day and documented the number of CVLs that were flagged as potentially eligible for removal. The investigator subsequently reviewed the EHR and documented the number of these potentially eligible devices that were removed within the next 24 hours. Devices identified as potentially eligible within 24 hours of a patient’s death were excluded for that day.

For the primary outcome, the average weekly proportion of potentially unnecessary CVLs removed, data was aggregated by calendar week (Monday through Sunday), calculated for the overall adult ICU population, and then stratified by clinician team for further analysis. Additional stratifications were performed, when indicated, for audit and feedback purposes. Weekly performance was selected to mitigate some of the expected daily variability in the number of potentially eligible devices. Data collection and analysis began on 9/5/2022 to obtain a performance baseline prior to implementation of the lists on 11/1/2022. Statistical process control (SPC) P charts, which display attribute data collected in subgroups of varying sizes for a measurable time, were used to compare the baseline period (September 2022–October 2022) and the intervention period (November 2022–August 2023) for the primary outcome for the PCCM-staffed (MICU, SMICU, MSICU) and the non-PCCM-staffed (CVICU, STICU, NSICU) ICUs. SPC G charts, which display the number of opportunities between incidences of rare events, were used to monitor the secondary outcome of days between CLABSIs for four distinct time periods for the PCCM-staffed and the non-PCCM-staffed ICUs: (1) preinitial COVID surge in South Carolina (September 2016–June 2020), (2) during peak of COVID in South Carolina (July 2020–February 2022), (3) post-COVID but preintervention (March 2022–October 2022), and (4) postintervention (November 2022–September 2023). Standard SPC chart rules [33] were utilized to detect signals of special-cause variation. SPC charts were created using QI Macros for Excel (v.2022.07, KnowWare International, Denver, CO, USA). In addition to these SPC charts, chi-square analysis was performed, comparing aggregated pre- and postintervention data for the proportion of potentially unnecessary CVLs identified and the proportion of these potentially unnecessary CVLs that were removed, and incidence rate ratios were calculated to compare CLABSI incidence during the four time periods outlined above.

### Continuous quality improvement

Postimplementation, the two PCCM physician investigators met monthly to review data. Performance feedback and additional education were provided at monthly multidisciplinary critical care meetings as needed. Targeted interventions for the PCCM-staffed teams were trialed and refined through iterative PDSA cycles (Table 2).

**Table 2.** Intervention timeline

Intervention Component	Date
Meetings with hospital leadership, CDSS steering committee, hospital informatics	March 2022 - April 2022
Development of inpatient lists	May 2022 - August 2022
Pre-intervention data collection	September 2022 - October 2022
Pre-intervention education	September 2022 - November 2022
ICU nursing and APP provider	September 2022 - November 2022
PCCM faculty and fellow education	September 2022 – October 2022
Adult ICU medical director and nurse manager education	October 2022
QI list roll-out	November 2022
Post-implementation audit and feedback and PDSA cycles	December 2022 - July 2023
**PCCM PDSA 1:** targeted audit and feedback at monthly PCCM fellow meetings	December 2022 - March 2023
**General Audit/Feedback (all ICU):** Critical Care Quality Assurance and Performance Improvement meetings	January 2023 - March 2023
**PCCM PDSA 2:** targeted audit and feedback at monthly PCCM faculty/APP meetings	January 2023 - July 2023

CDSS = clinical decision support system, ICU = intensive care unit, APP = advanced practice provider, PCCM = pulmonary/critical care medicine, PDSA = plan, do, study, act.

## Results

The initiative included 12 months of data, encompassing 3,426 CVL days across all adult ICUs. A timeline for the development and implementation of all components of the intervention is outlined in Table 2.

### All adult ICUs combined

**Figure 2.**
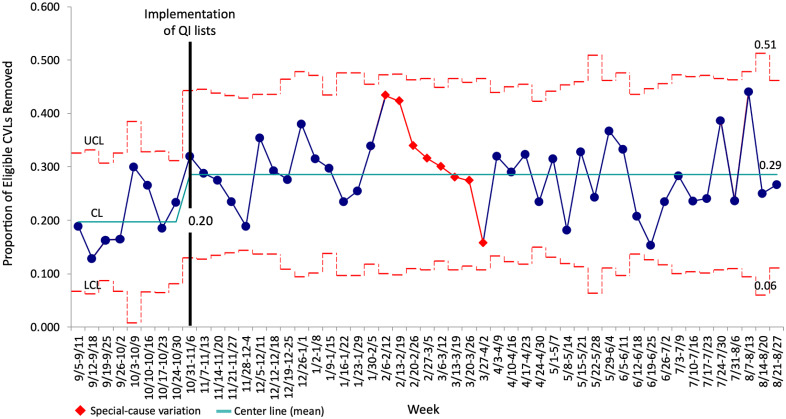
P chart - removal of eligible central venous lines (CVLs) within 24 hours over time, all adult ICUs. P chart demonstrating the weekly proportion of CVLs that were potentially eligible for removal that were removed within 24 hours across all adult intensive care units before and after implementation of quality improvement lists. The mean proportion (centerline, CL) is shown in teal, and the upper and lower control limits (UCL and LCL, ± 3 standard deviations from the CL) are depicted as dashed red lines. The red diamond markers/lines on the chart identify periods where special-cause variation was observed.

**Figure 3.**
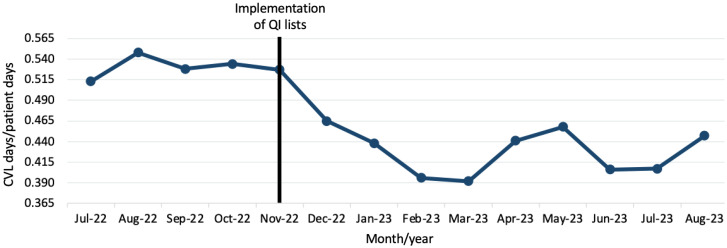
Changes in CVL utilization over time across all ICUs. CVL = central venous line, QI = quality improvement, ICUs = intensive care units.

In the preimplementation period (September-October 2022), 25.2% of 2,678 CVLs across all adult ICUs were flagged as potentially eligible for removal, and the mean weekly proportion of potentially eligible CVLs that were removed within 24 hours was 20.0%. Postintervention, the proportion of CVLs that were flagged as potentially eligible remained similar at 23.8% of 11,557 CVLs (*p* = 0.112). However, the mean weekly proportion of potentially eligible CVLs that were removed within 24 hours increased to 29% (*p* < 0.0001, Figure 2). The intervention was also associated with an overall decrease in CVL utilization from a mean of 0.53 CVL days/patient days in the 4 months prior to the intervention to 0.45 CVL days/patient days at the end of the data collection period (Figure 3). Finally, mean days between CLABSIs increased from 10.8 days in the 8 months prior to the intervention to 13.3 days postintervention (Supplemental Figure 2), though CLABSI incidence per 1,000 CVL days did not significantly change (incidence rate ratio 1.033, 95%CI 0.4855–2.222, *p* = 0.9295).

### PCCM-staffed ICUs vs. non-PCCM-staffed ICUs

#### CVL removal performance

**Figure 4.**
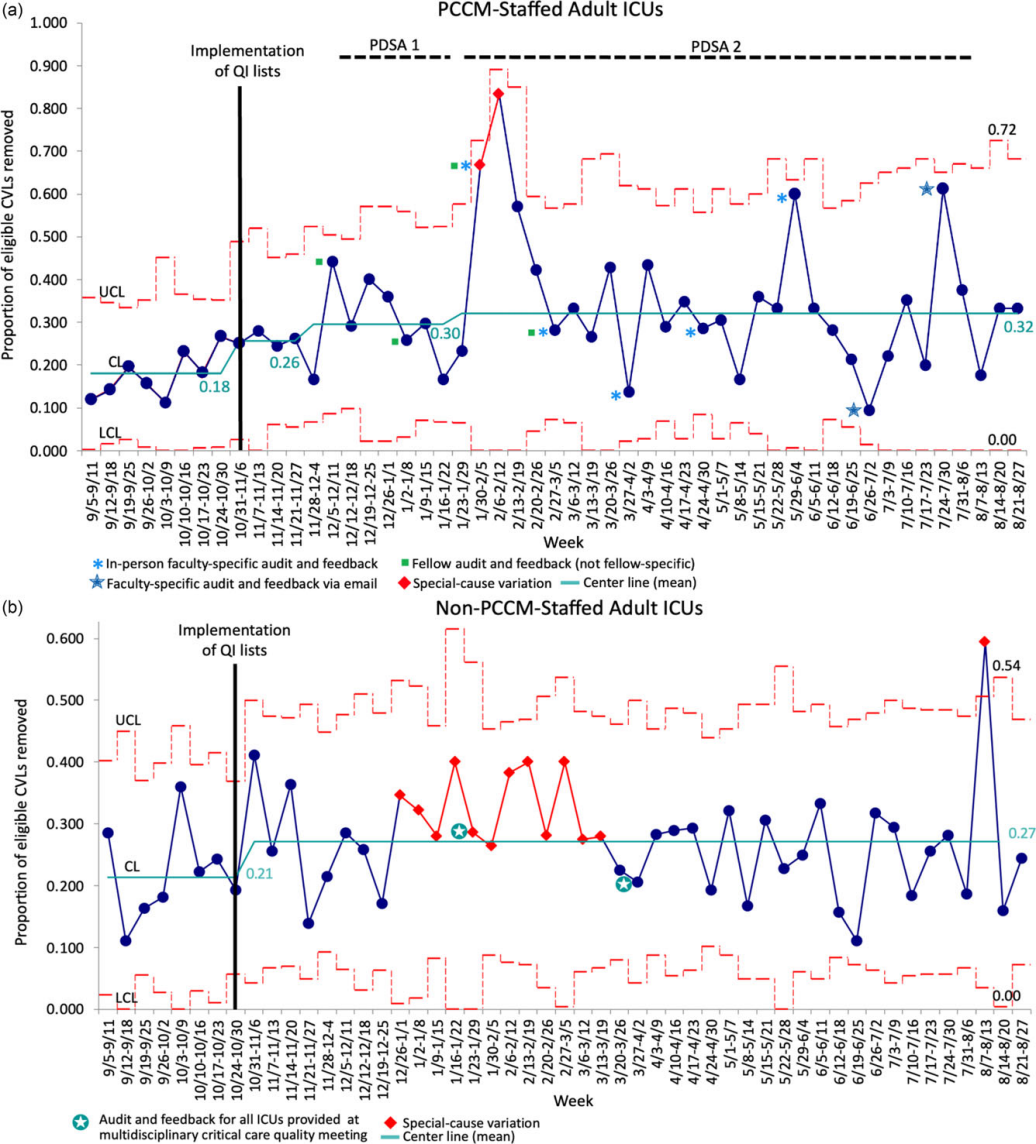
a: P chart - removal of eligible central venous lines (CVLs) within 24 hours over time in pulmonary/critical care medicine (PCCM)-staffed intensive care units (ICUs) in the context of the intervention and targeted plan, do, study, act (PDSA) cycles versus non-PCCM-staffed ICUs that did not perform iterative PDSA cycles. A: P chart demonstrating the weekly proportion of CVLs that were potentially eligible for removal that were removed within 24 hours in PCCM-staffed ICUs. b: P chart demonstrating the weekly proportion of CVLs that were potentially eligible for removal that were removed within 24 hours in non-PCCM-staffed ICUs. The mean proportion (centerline, CL) is shown in teal, and the upper and lower control limits (± 3 standard deviations from the CL) are depicted as dashed red lines. The red diamond markers/lines on the chart identify periods where special-cause variation was observed.

Preimplementation, 26.6% of CVLs in PCCM-staffed ICUs and 24.7% of CVLs in non-PCCM-staffed ICUs were flagged as potentially eligible for removal, and the mean weekly proportion of potentially eligible CVLs that were removed within 24 hours was 18% for PCCM-staffed ICUs and 21% for non-PCCM-staffed ICUs (Figure 4). In the initial postimplementation month (November 2022), this proportion significantly increased to 25.4% for PCCM-staffed ICUs (*p* = 0.025), while the increase to 26.8% for non-PCCM-staffed ICUs was not statistically significant (*p* = 0.155). Subsequently, using a quasi-experimental approach, two PDSA cycles were sequentially introduced for the PCCM-staffed ICUs (Table 2, Figure 4a). The first of these cycles, PDSA 1, was implemented in December 2022, continued through March 2023, and consisted of providing PCCM fellows with monthly performance comparisons between PCCM-staffed and non-PCCM-staffed ICUs. During the PDSA 1 interval prior to the introduction of PDSA 2, the mean weekly proportion of potentially eligible CVLs removed in PCCM-staffed ICUs further slightly increased to 30.3%. The second PDSA cycle, PDSA 2, targeting PCCM faculty was implemented in January 2023 and continued through July 2023. Monthly audit and feedback were provided in the form of public peer comparisons and ranking of specific attending physicians’ removal performance. During this intervention, the mean weekly proportion of potentially eligible CVLs removed incrementally increased to 32.3%. Though the magnitude of change in removal proportion was enough to raise the center line (mean) on the SPC charts, these increases were not statistically significant relative to the initial postimplementation month by chi-square analysis (*p* = 0.115 for PDSA 1 and *p* = 0.058 for PDSA 2). Notably, however, each episode of audit and feedback was associated with transient increases in the removal of potentially unnecessary CVLs during either the week of the feedback or the week immediately following, and the initiation of PDSA 2 was associated with special-cause variation. The correlation of the list intervention and audit and feedback with specific attending physician catheter removal performance is depicted in Supplemental Figure 3. There was variable association between audit and feedback and the proportion of potentially eligible CVLs removed, though lower baseline removal performance correlated with greater improvement after feedback (Supplemental Figure 3A). While the magnitude of change in removal performance also varied between attending physicians, all demonstrated improvement (Supplemental Figure 3B). The overall proportion of eligible CVLs removed for the entire postintervention period was significantly higher than that of the preintervention period (30.5% vs. 18.0%, *p* < 0.0001). Additionally, postintervention, the proportion of CVLs flagging as potentially eligible for removal decreased (20.1% vs. 26.6%, *p* < 0.0001).

Although the non-PCCM-staffed ICUs did not receive targeted interventions, leaders of all adult ICUs were provided with data on individual ICU performance compared with that of other ICUs in January and March 2023. There was a transient, special-cause variation to shift toward better removal performance between January 2023 and the beginning of March 2023, followed by a decrement in performance. However, at the end of the data collection period, the mean weekly proportion of potentially eligible CVLs removed in non-PCCM-staffed ICUs remained improved from baseline (27.1% vs. 21.2%, *p* = 0.026, Figure 4b). The overall proportion of CVLs flagging as potentially eligible for removal postintervention increased slightly (27.0% vs. 24.7%), though this increase was not statistically significant (*p* = 0.08).

#### CLABSI incidence

**Figure 5.**
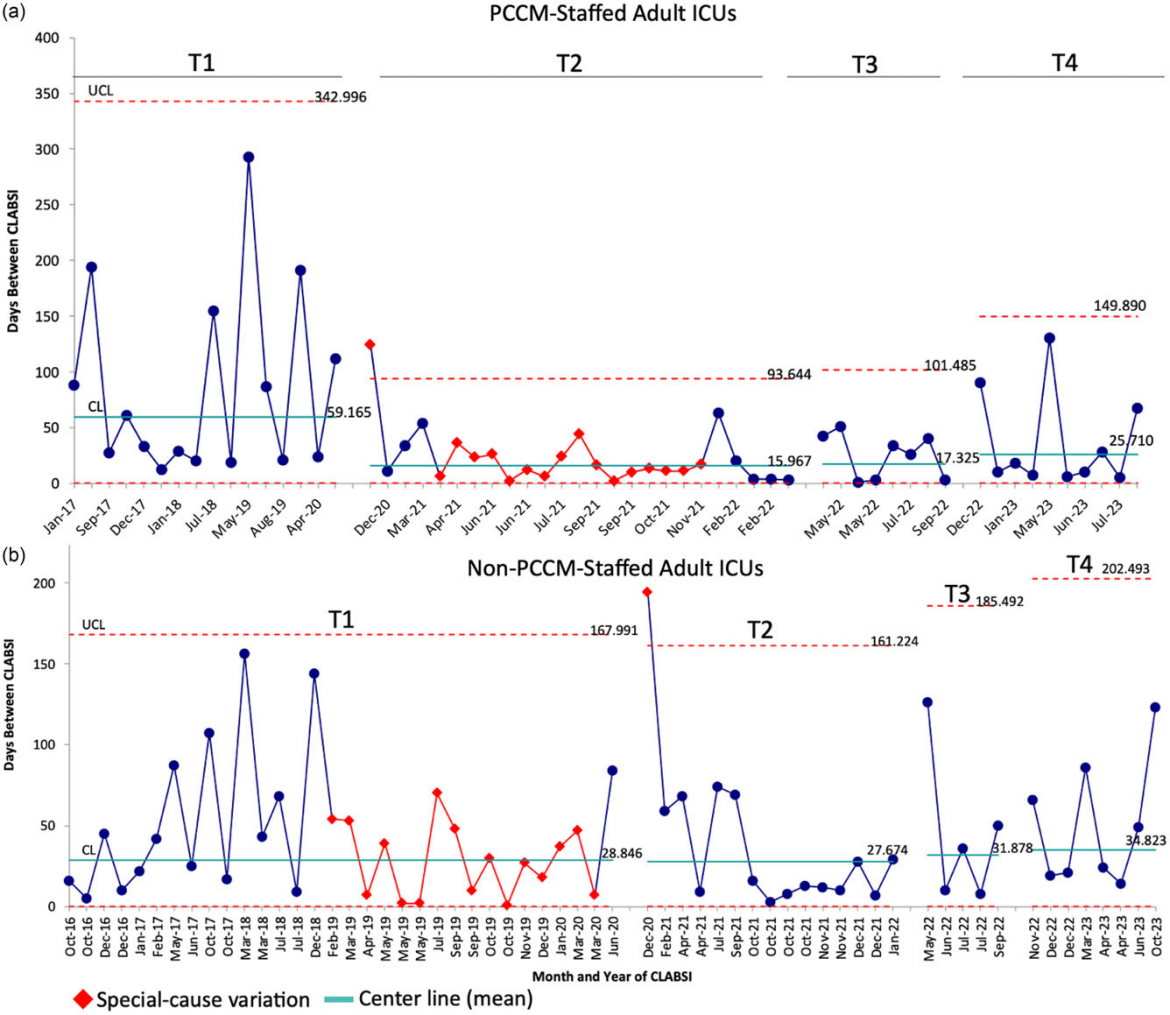
G chart – central line associated bloodstream infection (CLABSI) incidence over time (Days between CLABSIs) for pulmonary/critical care medicine (PCCM)-staffed adult intensive care units (ICUs) vs. Non-PCCM-staffed adult ICUs. A: G chart demonstrating days between CLABSI events for PCCM-staffed ICUs. B: G chart demonstrating days between CLABSI events for non-PCCM-staffed ICUs. The mean proportion (centerline, CL) is shown in teal, and the upper control limit (+ 3 standard deviations from the CL) is depicted as dashed red lines. The red diamond markers/lines on the chart identify periods where special-cause variation was observed. T1 represents the baseline, preinitial COVID surge in South Carolina (September 2016–June 2020). T2 represents the time during which South Carolina experienced multiple COVID surges (July 2020–February 2022). T3 represents the time interval following the last major COVID surge and prior to the intervention (March 2022–October 2022). T4 represents the postintervention period (November 2022–September 2023).

CLABSI incidence over time, in terms of days between CLABSIs, for PCCM-staffed vs. non-PCCM-staffed ICUs is presented in Figure 5. Prior to the COVID-19 pandemic (T1), CLABSIs occurred less frequently in PCCM-staffed ICUs (mean every 59.2 days, Figure 5a) than in non-PCCM-staffed ICUs (mean every 28.9 days, Figure 5b). During this period, non-PCCM-staffed ICUs experienced a trend of increasing frequency of CLABSIs which met criteria for special-cause variation on the SPC chart between February 2019 and March 2020, followed by a prolonged (6-month) CLABSI-free interval which also met criteria for special-cause variation. The COVID-19 pandemic (T2) was associated with a significant increase in CLABSI incidence for PCCM-staffed ICUs, with mean days between CLABSIs decreasing by 43.2–16.0. Conversely, mean days between CLABSIs in non-PCCM-staffed ICUs remained similar to the pre-COVID baseline (27.7 vs. 28.9 days). In the 8 months following the significant inpatient COVID-19 surges and prior to implementation of the intervention (T3), days between CLABSIs increased modestly in both ICU groups (17.3 vs. 16.0 days in PCCM-staffed ICUs; 31.9 vs. 27.7 days in non-PCCM-staffed ICUs). Finally, postintervention (T4), days between CLABSIs increased by a mean of 8.4 days in PCCM-staffed ICUs, and 2.9 days in non-PCCM-staffed ICUs. CLABSI incidence per 1,000 CVL days and incident rate ratios for PCCM-staffed ICUs and non-PCCM-staffed ICUs during these four distinct time periods are reported in Supplemental Appendix Tables 2 and 3. By this metric, the only statistically significant difference in CLABSI incidence occurred when comparing T2 vs. T1 for PCCM-staffed ICUs (incidence rate ratio 2.666, 95%CI 1.3591–5.3719, *p* = 0.002).

## Discussion

This CDSS-focused intervention was associated with an increase in timely removal of unneeded CVLs, concurrent decreases in CVL utilization, and increases in days between CLABSIs. The use of audit and feedback-focused PDSA cycles likely contributed to further incremental improvements in removal performance for PCCM-staffed ICUs, though the potential impact of each feedback session appeared to be transient. This initiative demonstrates how a multidisciplinary team can leverage CDS and the EHR to improve implementation of best practices and the quality of care delivered.

While the COVID-19 pandemic strained healthcare systems and illuminated weaknesses in safety culture, prepandemic studies also demonstrated the pervasiveness of unnecessary CVL use [34–36], with nearly one-third of active CVLs deemed unnecessary in one study [35]. Consistent with these findings, more than 25% of all CVLs in the present initiative were identified by the QI lists as potentially unnecessary during the preintervention period. As the development of CLABSIs is directly related to CVL utilization, it is critical that clinicians and hospitals identify strategies to reduce unnecessary CVLs. Implementation of checklists to prompt daily review of CVL necessity during patient rounds [36–40] has been one of the most commonly employed approaches. While use of checklists has correlated with some success in reducing CVL days and CLABSIs, these studies were performed prepandemic with better ICU staffing and a more experienced nursing workforce [9]. Even as staffing levels recover, checklist-focused interventions do not address many of the identified barriers to timely CVL removal, including the actual process of determining CVL necessity, a task which may be hampered by the cognitive and time burdens of reviewing many pieces of data for each patient. As patients grow increasingly complex over time [41], these burdens will continue to expand and may limit checklist effectiveness.

The current initiative sought to utilize principles of human factors engineering [29–31] and user-centered design [42] to develop an alternative to traditional checklists that could promote timely CVL removal while also lessening cognitive and time burdens. While the behavioral target of the EHR-embedded inpatient QI lists is similar to that of checklists, prompting daily consideration of CVL necessity, the QI lists offer multiple important advantages over traditional checklists. For one, they ensure that all active CVLs are identified without clinicians needing to remember to check for them at the bedside. This is important, as studies have demonstrated that clinicians are often unaware of the presence of CVLs [43]. The QI lists further streamline clinicians’ workflows and reduce time expenditures by automatically gathering relevant data from multiple areas within the EHR and presenting aggregated and instantaneously updated information for all patients on a single screen. Lastly, the integrated CDSS synthesizes the collected data and utilizes smart logic to provide clear, patient-specific removal recommendations, thus eliminating much of the cognitive load associated with this task.

While implementation of the QI lists was associated with improvements in the CVL metrics of interest, the mean proportion of potentially unnecessary CVLs that remained in place after 24 hours remained higher than desired. There are several potential contributors to this incomplete response. For instance, while the logic structure for the CDSS incorporates all objective indications for CVLs, more subjective reasons such as difficulty obtaining peripheral venous access and maintaining CVLs for comfort-oriented care during end-of-life care are neither clearly definable nor consistently queryable within Epic and therefore could not be included. Removal performance may have also been hindered by competing interests of care team members. Physicians and APPs may be hesitant to remove a CVL due to concerns that they may need to personally replace that CVL should a patient’s condition subsequently deteriorate, while nurses may have felt disincentivized to remove a reliable intravenous access that also afforded convenience for obtaining laboratory specimens. Finally, use of a passive CDSS, while less disruptive than an interruptive pop-up alert, nevertheless required clinicians to have awareness of the QI lists and to (1) deviate from their typical workflow to initially locate and save the QI lists and (2) adapt their daily workflow to review these specific lists. The utilization of audit and feedback was intended to partially mitigate these latter challenges [44] and was associated with transient, 1–2 week interval, improvements in removal performance, perhaps suggesting a need for more frequent feedback to drive continued prioritization of daily CVL review, particularly during early stages of implementation.

Importantly, this quality improvement initiative was associated with an increase in days between CLABSIs. An SPC G chart was employed to examine this measurement as it is a more sensitive method of observing early changes in the occurrence of rare events [45]. Although the more traditional metric of CLABSI incidence, CLABSI per 1,000 CVL days, did not significantly decrease, this observation is likely attributable to the overall rarity of CLABSI events relative to pooled CVL days and a relatively short postintervention period. Despite this rarity, healthcare quality doctrine takes a “zero tolerance” approach to CLABSIs, thus, we consider the early signal of CLABSI reduction demonstrated by the data presented here to be an important finding.

We acknowledge several important limitations to our intervention. First, as our institution was struggling with persistently high rates of CLABSIs, the QI lists were implemented rapidly after their development to limit ongoing patient harm. As a result of this need, the preintervention data collection period was relatively short when compared with the postintervention data collection period. Additionally, nurse staffing and capacity strain did improve during the course of this intervention, which may have contributed to the observed improvements in removal performance and CLABSI incidence. Finally, as a single-center initiative, our results may not be generalizable to other institutions. However, as nearly all US hospitals have an EHR, and Epic holds a significant plurality of both the US hospital market share and percentage of US beds [46], the general approach to the intervention could be replicated at many other centers.

## Conclusions

A significant proportion of active CVLs at our institution were potentially unnecessary. This implementation of science-informed EHR and CDSS-focused QI intervention was associated with sustained improvements in timely CVL removal with concurrent decreases in overall CVL utilization and CLABSIs. This initiative is an example of effective use of CDS to improve implementation of best practices. Future studies should focus on further improving workflow integration (e.g. displaying the QI lists on dedicated screens in ICUs or as the initial screen upon login to the EHR), determining the optimal cadence for providing audit and feedback, and the ability to assess generalizability through multicenter collaboration.

## Supporting information

Spiegel and Goodwin supplementary material Spiegel and Goodwin supplementary material

## Supplementary material

The supplementary material for this article can be found at https://doi.org/10.1017/cts.2024.566.
