# Supplementary material for: Development and implementation of a clinical decision support system-based quality initiative to reduce central line-associated bloodstream infections
Source: J Clin Transl Sci. 2024 Sep 23;8(1):e132. doi: 10.1017/cts.2024.566 (PMC11428117; doi:10.1017/cts.2024.566)
Supplement: Spiegel and Goodwin supplementary material [file S2059866124005661sup001.docx]

Supplemental Figure 1: Pre-Existing CVL Interruptive Alerts


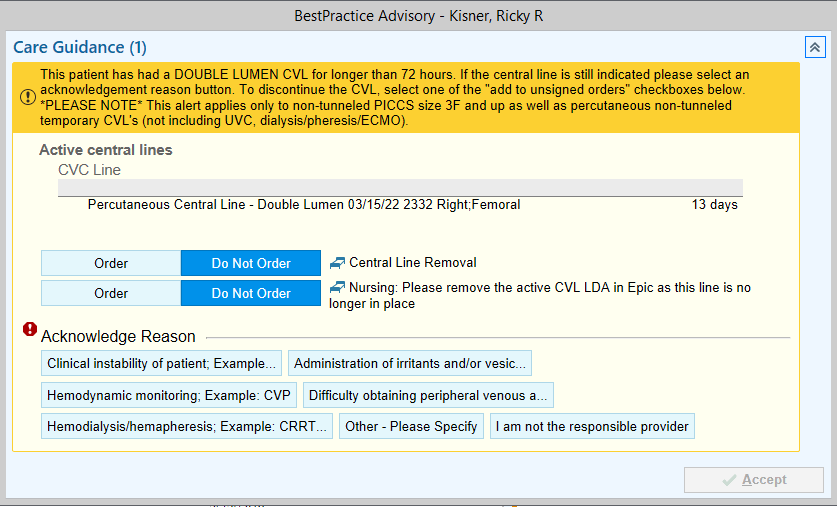


© 2022 Epic Systems Corporation

Supplemental Table 1: Application of the Quality Implementation Framework in Development and Implementation of the Intervention

| **Phase** | **Components** | **QI intervention** |
| --- | --- | --- |
| Phase 1: initial considerations regarding the host setting | Conducting needs and resources assessment   - What problems or conditions does the innovation address? - What parts of the organization and who in the organization will benefit from improvement efforts? | - Intervention addresses one contributor to the rising CLABSI incidence - Organization benefits from intervention as CLABSIs are associated with significant non-reimbursed costs - Patients benefit from the intervention as it aims to reduce CLABSIs which increase morbidity and mortality risk |
|  | Conducting fit assessment   - Does the innovation fit the setting? - How well does the innovation match the organization’s missions, priorities, values, and strategy for growth? | - Intervention designed to fit into clinician workflow (inpatient QI lists, no pop-up alerts) - Intervention aligns with institutional mission to improve quality and safety in healthcare |
|  | Conducting capacity/readiness assessment   - To what degree does the organization have the will and the means to implement the innovation? - Is the organization ready for change? | - Our institution has strong leadership and infrastructure dedicated to quality improvement, patient safety, and innovation - Our institution has suitable EHR (Epic) for clinical decision support interventions - Clinicians have access to computers on rounds to utilize intervention |
|  | Possibility for adaptation   - Should the planned innovation be modified in any way to fit the host setting and target group? - How will changes to the innovation be documented and monitored during implementation | - Intervention should apply to all adult ICUs - Daily Epic reports will be run to evaluate CVL de-escalation rates. Iterative changes to refine the logic will be made as needed, and date of change will be recorded by investigator |
|  | Obtaining explicit buy-in from critical stakeholders and fostering a supportive organizational climate   - Do we have genuine and explicit buy-in from leadership with decision-making power and from front-line staff? - Have we effectively dealt with important concerns, questions, or resistance to this innovation? What possible barriers to implementation need to be lessened or removed? - Can we identify and recruit innovation champion(s)? - How can the organization assist the champion in the effort to foster and maintain buy-in for change? | - Intervention presented to and favorably reviewed by executive leadership in multiple forums (CDSS steering committee, presentation to Performance Excellence Committee) - Biggest barrier to implementation is IS support for the EHR build. This is overcome with use of a physician builder to complete much of the build - Investigators serve as champions and ICU nurse managers and medical directors recruited as additional champions |
|  | Building general/organizational capacity   - What infrastructure, skills, and motivation of the organization need enhancement in order to ensure the innovation will be implemented with quality? | - Physician builder utilized and clinician stakeholders engaged to ensure optimal CDSS logic and that the CDSS is integrated into current workflow. Existing, ineffective CDS (pop-up alert based only on CVL days) needed to be de-implemented. - For pulmonary/critical care-run ICUs, the intervention was selected to be the annual QI project for the fellows and APPs |
|  | Staff recruitment/maintenance   - Who will implement the innovation? - Who will support the practitioners who implement the innovation? | - Intervention intended to include attending physicians, APPs, fellows, bedside nurses, and nurse managers. Residents and students able to participate but not universally included in training. - Investigator team available to provide support and troubleshooting |
|  | Effective pre-innovation staff training   - Can we provide sufficient training to teach the why, what, when, where, and how regarding the intended innovation? | - Pre-intervention training provided to ICU medical directors, nurse managers, and APPs in all units. In ICUs with pulmonary/critical care leadership, bedside nurses and fellows also received education. |
| Phase 2: Creating structure for implementation | Creating implementation teams   - Who will have organizational responsibility for implementation? - Can we develop a support team of qualified staff to work with front-line workers who are delivering the innovation? | - Investigators to lead the implementation and will be available for clinicians for troubleshooting issues. Nurse managers and ICU medical directors also able to provide support. |
|  | Developing an implementation plan   - Can we create a clear plan that includes specific tasks and timelines to enhance accountability during implementation? - What challenges to effective implementation can we foresee that we can address proactively? | - ICU medical directors and nurse managers educated on timeline for intervention roll-out - Initial uptake of new intervention expected to be the biggest challenge. Investigator team to provide frequent reminders and also audit and feedback on rates of eligible CVL removal |
| Phase 3: Ongoing structure once implementation begins | Technical assistance/coaching/supervision   - Can we provide the necessary technical assistance to help the organization and practitioners deal with the inevitable practical problems that will develop once the innovation begins? | - Physician builder for the intervention is one of the investigators, and is therefore able to provide the necessary technical assistance. IS also available for additional support |
|  | Process evaluation   - Do we have a plan to evaluate the relative strengths and limitations in the innovation’s implementation as it unfolds over time? (including data plan) | -Investigators to run reports daily to query the number of eligible CVLs and what proportion of these are removed by the next day |
|  | Supportive feedback mechanism   - Is there an effective process through which key findings from process data related to implementation are communicated, discussed and acted upon? - How will process data on implementation be shared with all those involved in the innovation? | PDSA cycles utilized to improve uptake after initial implementation   - Audit and feedback of eligible CVL removal performance performed and results reported at monthly leadership meetings (included all ICU medical directors and nurse managers as well as additional quality-focused ICU team members) - Pulmonary/critical care division received additional audit and feedback at the individual attending level and at monthly fellow meetings |
| Phase 4: Improving future applications | Learning from experience   - What lessons have been learned about implementing this innovation that we can share with others who have an interest in its use? | - Frequent check-ins/audit and feedback important for implementation; these check-ins identified some opportunities for improving logic structure of CDSS and comparing performance with that of other ICUs/attending physicians appeared to improve uptake |

QI = quality improvement, CLABSI = central line associated bloodstream infection, ICU = intensive care unit, CDSS = clinical decision support system, EHR = electronic health record, CVL = central venous line, APP = advanced practice provider, IS = information solutions, PDSA = plan, do, study, act

Supplement: Institution’s Peripheral Vasopressor Policy

# OVERVIEW

- Vasopressor medications are used in the critical care setting to increase blood pressure for improved organ perfusion in the setting of shock.
- Traditionally patients require a central venous catheter for administration of vasopressor medications regardless of dosing or duration due to risk of extravasation/tissue injury that could occur with use of a peripheral IV.
- Recent data shows that the utilization of a peripheral IV following proper protocol and precautions in patients requiring low dose vasopressor support can decrease time to treatment, improve patient outcomes, decrease healthcare costs due to decreased use of central venous access devices, and decrease the prevalence of central line associated blood stream infections (CLABSI) in the critical care setting.

# DEFINITIONS

## Low-Dose Vasopressors

- - **Norepinephrine:** Less than or equal to 10 mcg/min
  - **Phenylephrine:** Less than or equal to 80 mcg/min
  - **Dopamine:** Less than or equal to 5 mcg/kg/min
  - **Epinephrine:** Less than or equal to 0.025 mcg/kg/min
  - Vasopressin is not included due to being highly vesicant and extravasations require medication intervention due to skin necrosis.

## Peripheral IV

- - For the purpose of this guideline, the peripheral intravenous catheter should be 20-gauge or greater and be placed no lower than the middle of the forearm.
  - Ultrasound confirmation must be obtained by an ultrasound guided peripheral IV trained RN or a physician.

## Extravasation

- - Extravasation is the accidental infusion of vesicant fluid into the tissue.
  - The signs and symptoms of extravasation are swelling, discoloration, coolness, pain at the site, redness, and pain when flushing/infusing.

## Phentolamine

- - Strong alpha-adrenergic antagonist which causes vasodilation and improves perfusion in the setting of improperly infused vasopressor medications.

# INDICATIONS

- Hypotension with a systolic blood pressure (SBP) of less than 90 mmHg or a Mean Arterial Pressure (MAP) of less than 65 mmHg

# GUIDELINE

- The bedside nurse will administer the chosen vasopressor medication via the IV pump and through a peripheral IV into the patient.
- Only low-dose vasopressors (as defined above) can be used via peripheral IVs.
- Only standard (not maximum) concentrations of each medication can be used in a peripheral IV.
- Effectiveness of the use of vasopressor medications can be defined as the achievement of a SBP or MAP goal.
- If there is a need for peripheral vasopressors for longer than 1 hour, a specific physician order for peripheral use must be added to the vasopressor order in Epic.

# PROCEDURE

1. The physician will order the vasopressor medication of choice for the patient. As long as the medication dosing required is consistent with low doses as defined above, a peripheral IV can be used.
2. The bedside nurse will assess the IV site. If there is any redness, pain with flushing, or lack of blood return, the IV must not be used. The IV should be in the middle of the forearm or above, it should have brisk blood return, there should be no resistance when flushing and should not have any swelling or redness at the insertion site.
3. Placement must be confirmed by ultrasound by either an ultrasound guided peripheral IV trained RN or a physician.
4. The infusion can be started once all of the above is met. If the patient is requiring more than the dosing above, central line placement should be considered.
5. If the patient is requiring more than 1 vasopressor (even if low dose) or IV pushes of a vasopressor, then the licensed provider should place a central line.
6. The IV site will be assessed for any extravasation symptoms every 2 hours and documented in the patient’s electronic medical record.
7. The physician will re-assess the patient and consider if the continued use of the peripheral IV is appropriate every 24 hours or as needed. If the patient has continued need for vasopressor medications, then central line placement should be considered.
8. If there are any signs of extravasation, the physician must be notified and the proper treatment techniques started. Signs and symptoms include redness, swelling, and pain with infusion/flushing.
9. A second peripheral IV should be available at all times in case the infusing IV is unable to be used.

# TREATMENT OF EXTRAVASATION

- If extravasation is suspected, then the bedside RN will switch the infusion to the secondary IV and aspirate as much as possible from the original IV before removing the IV.
- Once the IV is removed, if no medical intervention is needed, a warm pack can be placed on the IV site.
- If medical intervention with Phentolamine is needed, follow pharmacy direction for subcutaneous injection around the IV site.
- Signs and symptoms include swelling, redness, pain at insertion site, blister formation, discoloration around the IV site, and pain when infusing/flushing the IV.


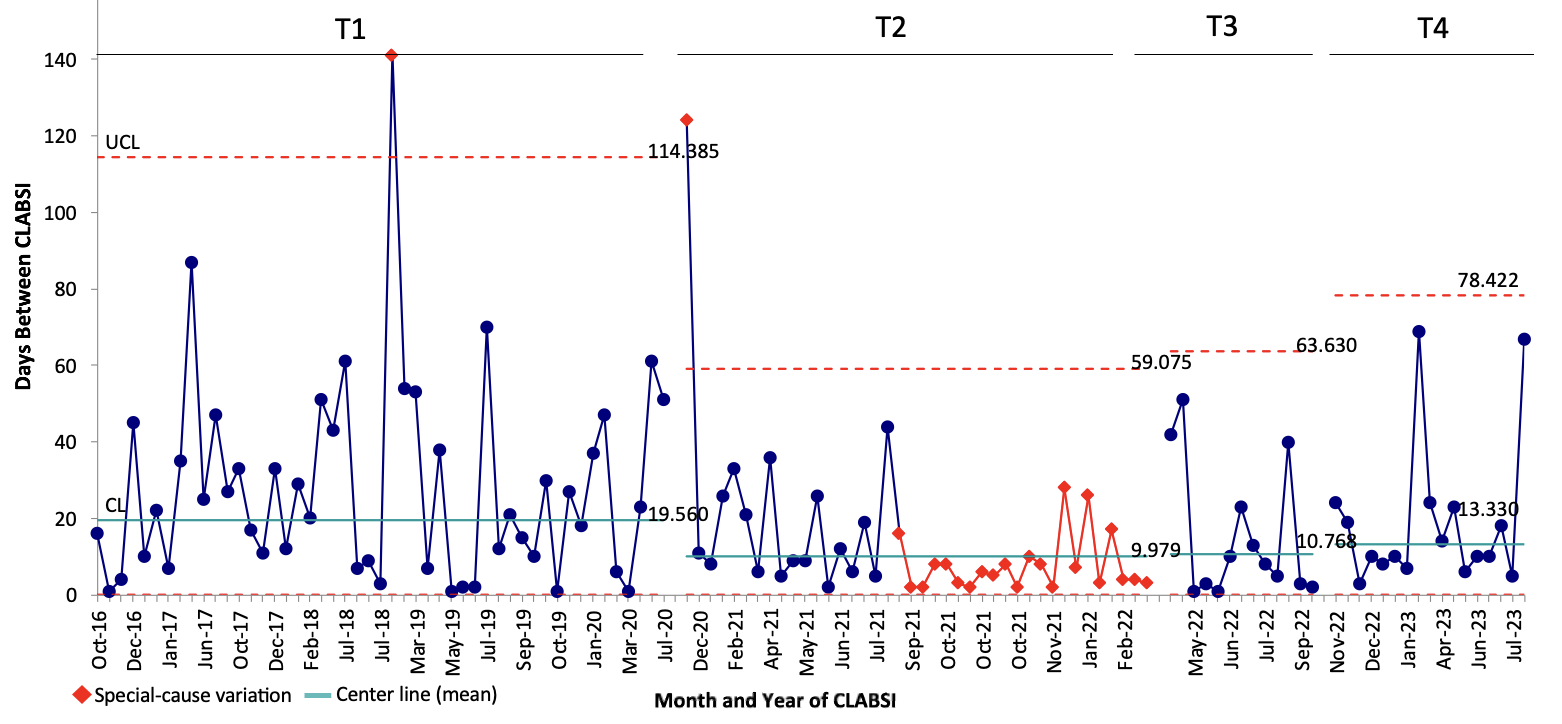
Supplemental Figure 2: G Chart- CLABSI Incidence Over Time (Days Between CLABSIs) for All Adult ICUs

Supplemental Figure 2. G-chart demonstrating days between central line-associated bloodstream infection (CLABSI) events for all adult intensive care units (ICUs). The mean proportion (centerline, CL) is shown in teal, and the upper control limit (UCL, + 3 standard deviations from the CL) is depicted as dashed red lines. The red diamond markers/lines on the chart identify periods where special-cause variation was observed. T1 represents the baseline, pre-initial COVID surge in South Carolina (September 2016-June 2020). T2 represents the time during which South Carolina experienced multiple COVID surges (July 2020-February 2022). Special cause variation was observed during T2, with increased incidence of CLABSIs observed during pandemic. T3 represents the time interval following the last major COVID surge and prior to the intervention (March 2022-October 2022). T4 represents the post-intervention period (November 2022-September 2023).

Supplemental Figure 3: The Association of Audit and Feedback on CVL Removal Performance by Individual Attending Physicians


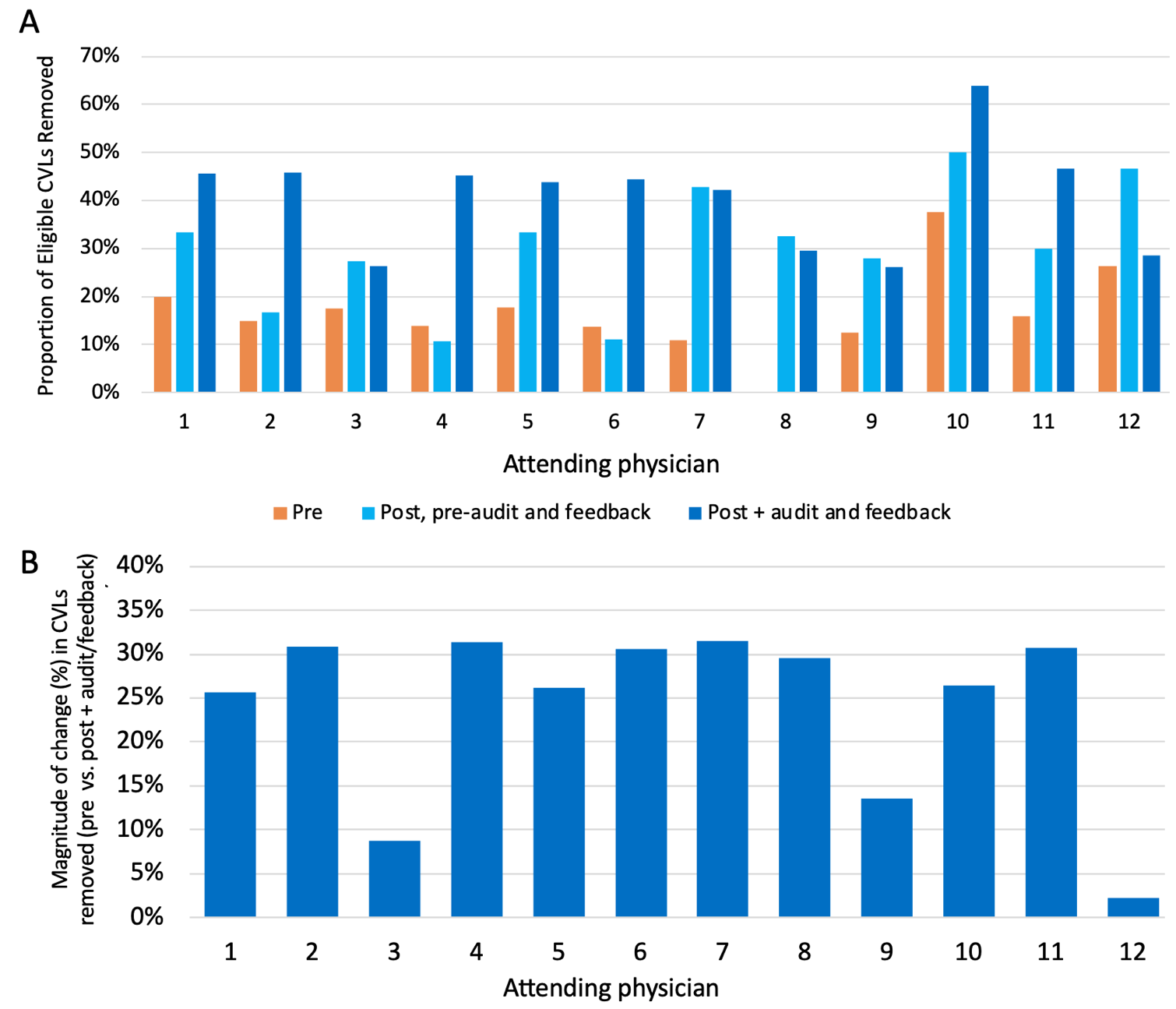


Supplemental Figure 3A: Central venous line (CVL) removal by attending physician pre-intervention, post-list implementation but prior to audit and feedback, and post-list implementation and with audit and feedback. Supplemental Figure 3B: Magnitude of change in CVL removal by attending physician between the pre-intervention and the post-intervention period with audit and feedback.

Supplemental Table 2: CLABSI Incidence Over Time

| Time Interval | Number of CLABSIs | Number of CVL days | CLABSIs/1000 CVL days |
| --- | --- | --- | --- |
| PCCM-Staffed Adult ICUs | | | |
| T1 | 16 | 22,645 | 0.707 |
| T2 | 24 | 12,738 | 1.884 |
| T3 | 9 | 5,199 | 1.731 |
| T4 | 9 | 5,843 | 1.540 |
| Non-PCCM-Staffed Adult ICUs | | | |
| T1 | 33 | 28,979 | 1.388 |
| T2 | 18 | 13,799 | 1.304 |
| T3 | 6 | 6,038 | 0.994 |
| T4 | 8 | 6,481 | 1.234 |

CLABSI = central line-associated bloodstream infections, CVL = central venous line, PCCM = pulmonary/critical care medicine, ICU = intensive care unit, T1 = July 2016 – June 2020, T2 = July 2020 – February 2022, T3 = March 2022 – October 2022, T4 = November 2022-August 2023

Supplemental Table 3: CLABSI Incidence Rate Ratios Over Time

| Time Interval Comparison | Incidence Rate Ratio | 95% Confidence Interval | P value |
| --- | --- | --- | --- |
| PCCM-Staffed Adult ICUs | | | |
| T2 vs. T1 | 2.666 | 1.3591 to 5.3719 | 0.002 |
| T3 vs. T2 | 0.919 | 0.3758 to 2.0475 | 0.851 |
| T4 vs. T3 | 0.890 | 0.3129 to 2.5300 | 0.807 |
| T4 vs. T2 | 0.818 | 0.3344 to 1.8218 | 0.624 |
| Non-PCCM-Staffed Adult ICUs | | | |
| T2 vs. T1 | 1.146 | 0.6073 to 2.0944 | 0.637 |
| T3 vs. T2 | 0.762 | 0.2475 to 2.0033 | 0.586 |
| T4 vs. T3 | 1.242 | 0.3780 to 4.3435 | 0.701 |
| T4 vs. T2 | 0.9463 | 0.3560 to 2.2872 | 0.918 |

CLABSI = central line-associated bloodstream infections, CVL = central venous line, PCCM = pulmonary/critical care medicine, ICU = intensive care unit, T1 = July 2016 – June 2020, T2 = July 2020 – February 2022, T3 = March 2022 – October 2022, T4 = November 2022-August 2023
